# Prevalence of Foot At-Risk and its Associated Characteristics among Outpatients with DiabetesMellitus in a Peruvian Public Hospital

**DOI:** 10.1900/RDS.2022.18.1

**Published:** 2022-03-31

**Authors:** Marlon Yovera-Aldana, Sonia Pérez-Cavero, Isabel Pinedo-Torres, Carlos Zubiate-López

**Affiliations:** 1 Neurosciences, Clinical Effectiveness, and Public Health Research Group, Universidad Cientifica del Sur, Lima, Peru,; 2 Endocrinology Service, Department of Medicine, Hospital Maria Auxiliadora, Lima, Peru,; 3 Endocrinology Service, Department of Medicine and Office for Teaching Support and Research, OADI, Hospital Daniel Alcides Carrion, Callao, Peru,; 4 Clinical and Health Efficacy Network, REDECS, Lima, Peru

**Keywords:** diabetes, diabetic foot, risk, IWGDF criteria, primary prevention

## Abstract

**OBJECTIVE:**

To assess the prevalence of patients at risk of developing diabetic foot complications (i.e. foot at-risk) and its clinical components according to the updated International Working Group on Diabetic Foot (IWGDF) criteria and to describe demographic and diabetes-related characteristics.

**METHODS:**

We conducted a cross-sectional study at María Auxiliadora Hospital between 2017 and 2018. The criteria for foot at-risk in the IWGDF 2019 risk stratification system are classified into four risk categories, R0-R3, ranging from no peripheral arterial disease (PAD) and no peripheral neuropathy (PN) to the presence of PAD or PN in combination with previous foot ulcer, amputation, or end- stage renal disease (R3). According to this system, we obtained prevalence ratios (PR) of foot at-risk categories dependent on sex, age, diabetes duration, and Total Symptom Score. A sample size of 402 subjects was included in the study.

**RESULTS:**

Subjects included had a mean age of 61 years, and 66% were female. There were no patients with type 1 diabetes, and 59% percent had adiabetes duration of less than ten years. The prevalence of foot at-risk was 54.3% defined by the IWGDF 2019 criteria, which gave prevalence17% higher than that defined with the previous 1999 criteria. PN and PAD frequency was 37.3% and 30.1%, respectively. Foot at-risk prevalence was 40% higher in those with severe Total Symptom Score (PR 1.40, 95% CI 1.09-1.80) and also 39% higher in men than in women (PR 1.39, 95% CI 1.17-1.64). Likewise, diabetes duration of more than ten years had a 25% higher prevalence of foot at-risk (PR 1.25, 95% CI 1.05-1.49), and those older than 60 years had a 20% higher presence of this condition (PR 1.20, 95% CI 1.0011.43).

**CONCLUSIONS:**

Our hospital faces a substantial burden of diabetic foot risk in men, patients with long diabetes duration, and those with painful neuropathy. More initiatives are required at primary or hospital level to detect this critical condition. Likewise, reference centers with multidisciplinary teams to apply prevention and therapeutic interventions are urgently needed.

## 1 Introduction

Diabetic foot complications significantly burden public health provision because of the suffering and disability of patients and the direct and indirect costs associated with this condition [1]. The prevalence of diabetic foot ulcers in high- income countries ranges from 8% to 15%, and 85% of amputations are preceded by an ulcer [2]. Despite the magnitude of the problem, few prevention activities are undertaken to reduce the disease burden at primary care health centers [3].

In 1999, the International Working Group ofDiabetic Foot (IWGDF) recommended to classify ulceration risk by category, and to educate patients in conducting self-care [4]. This classification categorizes patients at risk of critical diabetic foot complications into four categories, ranging from 0 to 3 (i.e. R0, R1, R2, R3), with each category predicting ulcer occurrence of 5.1%, 14.3%, 18.8%, and 55.8%, respectively, at three years of follow-up [5]. Many countries have implemented detection systems based on this initiative at both primary and hospital level [6-13]. There are other systems that include almost all the clinical components [14-16], which are useful to determine patients at risk of foot problems [17].

In order to predict the occurrence of ulceration accurately, some authors have recommended subdividing IWGDF categories 2 and 3 because they contain heterogeneous factors [18]. However, a recent systematic review concluded that PAD had the same risk as PN [19], which resulted in the modification of the criteria after they had been established for 20 years to prevent misclassification and its consequent poor follow-up, especially in those under diagnosed [20].

Many prevention programs worldwide use the previous 1999 criteria, but no series has been published to date that employs the new 2019 criteria that followed the recent update. There is a need to evaluate the possible change in the prevalence of diabetes patients at risk of foot ulceration by means of the new IWGDF criteria [21]. Therefore, we aimed to reassess this prevalence and its clinical consequences as well as to describe the demographic and diabetes-related characteristics in outpatients in a diabetic foot unit of a Peruvian hospital.

## 2 Materials and methods

### 2.1 Study design

A cross-sectional study was conducted at María Auxiliadora Hospital, Lima, Peru, during 2017 and 2018. In this period, approximately 2,500 patients with diabetes mellitus and low income from the southern region of Lima City attended the endocrinology department at this hospital per year. The diabetic foot at-risk program began in 2015; it offered a prophylactic examination of lower limb injuries in diabetic outpatients. At their first endocrinology consultation during the year all patients were scheduled for a foot at-risk screening.

### 2.2 Population sample

We included subjects who had their first medical consultation during the recruitment period. Patients with active foot ulcers and conditions that made it difficult to assess the foot at-risk category correctly were excluded from the study. These conditions included:

- Hearing loss
- Cognitive impairment
- Linguistic barriers
- Venous insufficiency
- Leg ulcer
- Toe amputation
- Acute infection
- Incomplete data

Based on a sample size of 402 subjects and assuming an expected proportion of foot at-risk patients of 50%, we calculated a confidence level of 95%, precision rate of 5%, and loss rate of 5%. We included all accessible populations in the analysis.

### 2.3 Clinical evaluation

An expert-validated form was used for the endocrinology staff at Maria Auxiliadora hospital according to the Delphi method. The form contained data from clinical and epidemiological history and physical examination of foot at-risk components according to 1999 IWGDF guidelines. Two endocrinologists performed clinical evaluations with good interobserver agreement.

The 1999 IWGDF criteria classify patients into four groups:

1. Low risk (R0): no peripheral neuropathy (PN).
2. Moderate risk (R1): only PN.
3. High risk (R2): PAD or deformity +/- PN.
4. Very high risk (R3): ulcer or amputation history.

The 2019 IWGDF criteria also classify patients into four groups, but the definitions of the categories differ from the 1999 criteria:

1. Low risk (R0): no PAD and no PN.
2. Moderate risk (R1): PAD or PN.
3. High risk (R2): PAD or deformity, PN + deformity, PAD + PN.
4. Very high risk (R3): PAD or PN with one of the following: a previous ulcer or amputation, or end-stage chronic kidney disease [22].

The investigators reclassified patients with the same data to obtain updated classification. Both classifications were employed.

We defined PN as an alteration in two or more neurological tests such as monofilament, 128 Hz tuning fork, and Achilles reflex. Monofilament was applied to the first toe and the first, third, and fifth metatarsal head. The 128 Hz tuning fork test was performed at the interphalangeal joint of the hallux. Regarding Achilles reflex, we evaluated whether there was a reflex absence or slow relaxation phase. We defined PAD as a pulse absence or Ankle Brachial Index (ABI) < 0.9 in any of the following arteries (posterior tibial and pedial, left and right). Patients with arterial calcification (ABI ≥ 1.3) in only some of the arteries were not included in the PAD group. We evaluated four deformities: flat foot, pes cavus, claw/hammertoes, and hallux valgus [22]. Type of symptoms, frequency, and intensity of neuropathy were evaluated using the Total Symptom Score [23,24]. The following scores and related symptoms were considered:

- 0: no symptoms
- 1 to 4.99: mild symptoms
- 5 to 9.99: moderate symptoms
- 10 to 14.99: severe symptoms

To assess foot care education, we verified whether previous foot care counseling (from any health professional) had been received or whether patients had acquired knowledge through other means (some knowledge of foot care). Regarding foot care habits, we explored whether patients had proper foot hygiene (clean feet during inspection), nail trimming (straight cut), and proper footwear (wide shoes, no internal seams with cushioning sole). We evaluated pre-ulcerative foot lesions as ungual mycosis, xerosis, limb hair, dorsal and plantar heloma, or interdigital mycosis. The test was considered positive if any injury was present in one of the limbs.

Regarding treatment, patients were classified into three groups, as follows:

1. Dietary management only
2. Oral antidiabetic drugs only
3. Insulin (with or without oral antidiabetic drugs)

### 2.4 Statistical analysis

We described foot at-risk frequencies and their categories according to the 2019 and 1999 IWGDF criteria, respectively, and the clinical findings of PN, PAD, and biomechanical deformity [22]. In bivariate analysis, we described demographic and clinical characteristics according to the 2019 classification. Pearson’s chi-square test was used to evaluate the association of categoric variables. According to the normality evaluation by the Shapiro-Wilk test for numeric variables, we performed one-way ANOVA or the Kruskal-Wallis test.

In multivariate analysis, we considered a generalized linear model with robust variance, logarithm link, and Poisson distribution, and we obtained crude prevalence rates (PR) for foot at-risk and their 95% confidence intervals according to age, sex, diabetes duration, instruction level, diabetes medication, and Total Symptom Score. We also performed an adjusted model with the same variables [25].

We analyzed the database with STATA version 15.1 and considered a significance level of 0.05.

### 2.5 Ethics

The María Auxiliadora Hospital Institutional Review Board approved our research protocol, and we followed the principles of the Helsinki Declaration. We did not evaluate subjects directly, but only reviewed clinical records. Names and personal ID numbers were hidden in the database.

## 3 Results

**Figure 1.**
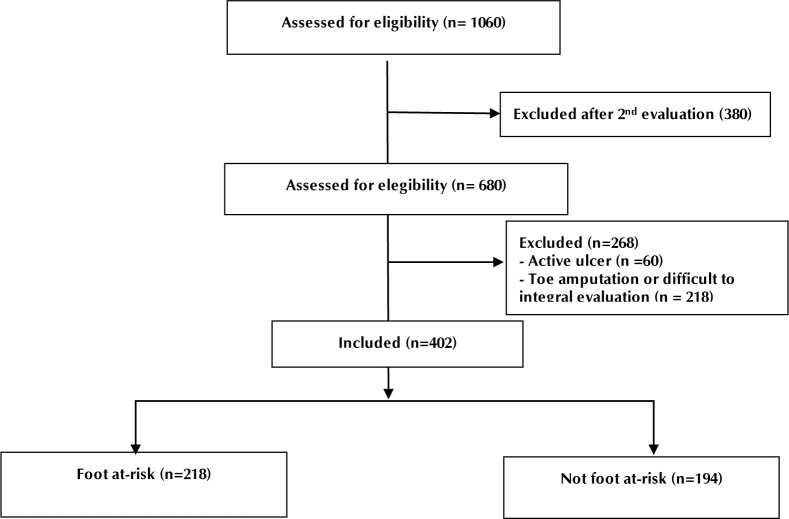
Flow chart of the selection of clinical records for the foot at-risk re-evaluation. Between January 2017 and December 2018, 1060 clinical files from foot at-risk patients, corresponding to 680 patients, were evaluated. Patients with active ulcers (60 subjects) and those with conditions that impeded a complete evaluation (218 subjects) were excluded from the study. Eventually, 402 patients and their clinical files from their first consultation were included in the analysis.

Between January 2017 and December 2018 we evaluated1060 foot at-risk forms corresponding to 680 patients. We excluded patients with active ulcers (60 subjects) and those with conditions that impeded a complete evaluation (218 subjects). Finally, in the analysis, we included 402 patients and their clinical files from their first consultation (**Figure 1**).

Our sample had a mean age of 61 years, and 66% of the subjects were women. We found no type 1 diabetes mellitus patients. The subjects had a median diabetes duration of 7 years, and 45% were users of insulin alone or in combination with oral antidiabetic drugs. Almost half of the patients (46.5%) had not received any information on foot care, and 70% presented inappropriate footwear during the consultation. About 75% presented some symptoms in the lower limbs, such as lancinating pain, tingling, burning, or numbness (**Table 1**).

**Table 1.** Clinical and demographic characteristics of patients who attended the foot at-risk program

Clinical characteristic	n (%)
Age (yr)	
Mean ± SD (years)	61 ± 11
<50	58 (14.4)
50- 59	120 (29.9)
60 -69	125 (31.1)
>70	99 (24.6)
Gender	
Male	140 (34.8)
Female	262 (65.2)
Education level	
Illiterate	18 (4.5)
Elementary	148 (36.8)
Highschool	194 (48.3)
College	42 (10.5)
Diabetes duration (yr)	
Median (IQR)	7 (3 to 13)
<10 years	239 (59.5)
10 -10.9 years	11 (27.6)
>20 years	52 (12.9)
Diabetes medication	
Only diet	22 (5.5)
Only oral antidiabetic drugs	196 (48.8)
Insulin + ADO	184 (45.7)
Foot care education	
Some knowledge about foot care	308 (76.6)
Previous foot care counseling	187 (46.5)
Foot care habits	
Proper foot hygiene	249 (61.9)
Proper nail trimming	175 (42.3)
Proper footwear	278 (69.2)
Total Symptom Score	
Median (IQR)	2.33 (1 to 5)
Absent	95 (23.6)
Mild (1-4.99)	204 (50.8)
Moderate (5-9.99)	95 (23.6)
Severe (10-14.99)	8 (1.9)
Pre-ulcerative foot lesions	
Ungual mycosis	279 (69.4)
Xerosis	240 (59.7)
Limb hair absent	228 (56.7)
Plantar heloma	208 (51.7)
Interdigital mycosis	161 (40.1)
Dorsal heloma	88 (21.9)
Foot at-risk components	
Peripheral neuropathy^a^	150 (37.3)
Peripheral arterial disease^b^	121 (30.1)
Deformity^c^	220 (54.3)
Previous ulcer	51 (12.7)

**Legend:**^a^Diagnosed if two or more tests were altered, including monofilament (first toe; first, third, and fifth metatarsal heads), 128 Hz tuning fork at the interphalangeal joint of the hallux, and Achilles reflex in the kneeling position.

^b^Diagnosed if the pulse was absent or Ankle Brachial Index (ABI) < 0.9 in any of the following arteries: posterior tibial and pedial, left and right.

^c^Diagnosed by the presence of flat foot, pes cavus, claw/hammer toes, or hallux valgus. *Abbreviations:* ADO - oral antidiabetic drug, IQR - interquartile range, PAD -peripheral artery disease, SD - standard deviation.

Regarding foot at-risk assessment, 54.3% had a moderate, high, or very high risk of foot complications according to the new IWGDF 2019 criteria; 17% more than with the 1999 criteria. PN was found in 37.3% and PAD in 30.1% of all patients (**Table 2**).

**Table 2.** Foot at-risk prevalence in outpatients with diabetes mellitus

Risk level	2019 IWGDF criteria	n (%)	1999 IWGDF criteria	n (%)
R0 (low)	No PN, no PAD	184 (45.7)	No PN	252 (62.6)
R1 (moderate)	PN or PAD	65 (16.2)	PN	36 ( 9.0)
R2 (high)	PN and PAD PN and deformity PAD and deformity	115 (28.6)	PN and PAD PN and deformity	78 (19.4)
R3 (very high)	PN or PAD and -Previous ulcer or -Previous amputation or -ESCKD	38 ( 9.5)	PN and -Previous ulcer or -Previous amputation	36 ( 9.0)
Foot at-risk	R1-R3	218 (54.3)	R1-R3	150 (37.4)

**Legend:** Diagnosis of PN if two or more tests were altered, including, including monofilament (first toe; first, third, and fifth metatarsal heads), 128 Hz tuning fork at the interphalangeal joint of the hallux, and Achilles reflex in the kneeling position. Diagnosis of PAD if the pulse was absent or Ankle Brachial Index (ABI) < 0.9 in any of the following arteries: posterior tibial and pedial, left and right. Diagnosis of deformity by the presence of flat foot, pes cavus, claw/hammer toes, or hallux valgus. *Abbreviations:* ESCKD - end-stage chronic kidney disease, IWGDF - International Working Group on the Diabetic Foot, PAD -peripheral artery disease, PN - peripheral neuropathy.

We found that 55% had some type of deformity; the most common were claw/hammer toe (30%), hallux valgus (25%), and flat foot (13%). Regarding the neurosensory tests, 35% showed alterations in the monofilament test, 32% in the 128Hz tuning fork test, and 32% in the Achilles reflex test. In the vascular examination, 21% had an altered pulse, and 35% had an ABI < 0.9 (**Table 3**).

**Table 3.** Clinical findings from the foot at-risk evaluation (n=402).

Foot at-risk component	Characteristic	n (%)
Biomechanical deformity		
Type of deformity	Claw toes	121 (30.1)
	Hallux valgus	100 (24.9)
	Flat foot	52 (12.9)
	Cavus foot	24 (6.0)
Number of deformities per	None	182 (45.3)
subject	1	158 (39.3)
	2	48 (11.9)
	3	13 (3.2)
	4	1 (0.3)
Peripheral neuropathy		
Semmes-Weinstein	Normal: 8 zones	261 (64.9)
monofilament measurement	Decreased: 1-7 zones	104 (25.9)
	Absent : 0 zones	37 ( 9.2)
Turning fork 128 Hz	Normal : > 10 s	233 (67.4)
	Decreased: <10 s	122 (31.2)
	Absent	36 ( 9.2)
Achilles reflex	Normal	267 (68.3)
	Reinforced	100 (25.5)
	Absent	24 ( 6.2)
Sensory tests showing altered	None	187 (46.5)
sensation by subject	1	65 (16.2)
	2	73 (18.2)
	3	77 (19.2)
Peripheral arterial disease		
Decreased or absent pulse	Right pedia	72 (18.4)
	Right posterior tibial	130 (33.2)
	Left pedia	76 (19.2)
	Left posterior tibial	136 (34.5)
Number of altered pulses per	None	318 (79.1)
patient	1 or more	84 (20.9)
Arteries with ABI >1.3 per	None	296 (83.8)
patients	One or more	57 (16.2)
Arteries with ABI <0.9 per	None	191 (64.5)
patients	1	56 (18.9)
(excluding patients with	2	32 (10.8)
calcification)	3	4 (1.4)
	4	13 (4.4)
Type of artery with ABI <0.9	Right pedia	53 (17.0)
(excluding results with	Right posterior tibial	36 (13.0)
calcification)	Left pedia	51 (15.9)
	Left posterior tibial	44 (15.9)
PAD, according to ABI	Normal (0.9 -1.29)	191 (54.1)
	Mild (0.70 -0.89)	79 (22.4)
	Moderate (0.50 - 0.69)	22 (6.2)
	Severe: (< 0.50)	4 (1.1)
	Calcification (> 1.3)	57 (16.2)

**Legend:** ABI -ankle-brachial index, PAD - peripheral artery disease.

In the low-risk group, 51% had deformities and 7% previous ulcers, but no PN or PAD. In the very high-risk group, 95% had PN, 60% PAD, and 31% a deformity (**Table 4**).

**Table 4.** Association between clinical characteristics and foot at-risk categories (n=402)

Clinical characteristic n (%)			Risk level		
	R0 (n=184)	R1 (n=65)	R2 (n=115)	R3 (n=38)	p-value
Age					
Mean ± SD (years)	59.1 ± 11	61.2 ± 9.9	63.7 ± 11.2	61.3 ± 11.9	0.630
<50	33 (18)	11 (17)	11 (10)	3 (8)	0.120
50- 59	59 (32)	17 (26)	27 (23)	17 (45)	
60 -69	56 (30)	21 (32)	39 (34)	9 (24)	
>70	36 (20)	16 (25)	38 (33)	9 (24)	
Gender					
Male	49 (27)	22 (34)	48 (42)	21 (55)	0.002
Female	135 (73)	43 (66)	67 (58)	17 (45)	
Education level					
Illiterate	7 (4)	3 (5)	7 (6)	1 (3)	0.370
Elementary	64 (35)	20 (31)	52 (45)	12 (32)	
High-school	89 (49)	36 (56)	49 (42)	20 (53)	
College	24 (13)	6 (9)	7 (6)	5 (12)	
Diabetes duration					
Median (IQR)	5 (3 to 10)	6 (3 to 15)	8 (4 to 15)	13.5 (5 to 20)	<0.001
< 10 years	125 (68)	39 (60)	61 (53)	14 (36)	0.001
10 -10.9 years	45 (24)	19 (29)	35 (30)	12 (32)	
>20 years	14 (8)	7 (11)	19 (17)	12 (32)	
Diabetes medication					
Diet	11 (6)	4 (6)	6 (5)	1 (3)	0.180
Oral antidiabetic drugs	99 (54)	34 (52)	44 (38)	19 (50)	
Insulin	74 (40)	27 (42)	65 (56)	18 (47)	
Foot care education					
Knowledge about foot care	144 (78)	51 (79)	86 (75)	27 (71)	0.730
Previous foot counseling	88 (48)	26 (40)	48 (42)	25 (66)	0.050
Foot care habits					
Proper foot hygiene	65 (65)	37 (57)	72 (63)	21 (55)	0.570
Proper nail trimming	80 (44)	27 (42)	46 (40)	17 (45)	0.920
Proper footwear	51 (28)	14 (21)	41 (36)	12 (32)	0.210
Total symptom score					
Median (IQR)	1.83 (0 to 4.66)	2.6 (0 to 4.66)	3 (1.66 to 5.32)	2.83 (2 to 5.99)	0.001
Absent	53 (29)	20 (31)	18 (16)	4 (11)	0.004
Mild (1-4.99)	39 (21)	6 (9)	14 (13)	5 (13)	
Moderate (5-9.99)	27 (15)	11 (17)	21 (18)	10 (26)	
Severe (10-14.99)	65 (35)	28 (43)	62 (54)	19 (50)	
Preulcerative lesions					
Ungual mycosis	126 (69)	48 (74)	78 (68)	27 (71)	0.840
Xerosis	110 (60)	39 (60)	70 (61)	21 (55)	0.940
Limb hair absent	109 (59)	37 (57)	61 (53)	21 (55)	0.760
Plantar heloma	92 (50)	36 (56)	62 (54)	18 (47)	0.780
Interdigital mycosis	67 (37)	19 (29)	57 (50)	18 (48)	0.020
Dorsal heloma	35 (19)	9 (14)	32 (28)	12 (32)	0.050
Foot at-risk components					
Peripheral neuropathy^a^	0 (0)	36 (56)	78 (68)	36 (94)	<0.001
Peripheral arterial disease^b^	0 (0)	29 (45)	80 (70)	12 (32)	<0.001
Deformity^c^	94 (51)	0 (0)	103 (90)	23 (60)	<0.001
Previous ulcer	13 (7)	0 (0)	0 (0)	38 (100)	<0.001

**Legend:**^a^Diagnosed if two or more tests were altered, including monofilament (first toe; first, third, and fifth metatarsal heads), 128 Hz tuning fork at the interphalangeal joint of the hallux, and Achilles reflex in the kneeling position.

^b^Diagnosed if the pulse was absent or Ankle Brachial Index (ABI) < 0.9 in any of the following arteries: posterior tibial and pedial, left and right.

^c^Diagnosed by the presence of flat foot, pes cavus, claw/hammer toes, or hallux valgus. *Abbreviations:* ADO - oral antidiabetic drug, IQR - interquartile range, PAD - peripheral artery disease, SD - standard deviation.

In multivariate analysis, foot at-risk prevalence was 40% higher in those with a moderate to severe Total Symptom Score (PR 1.40, 95% CI 1.07-1.82, p=0.01), and it was 39% higher in men than in women (PR 1.39, 95% CI 1.17-1.64, p=0.001). Likewise, patients with a diabetes duration of more than ten years had a 25% higher prevalence of foot at-risk (PR 1.25, 95% CI 1.05-1.49, p=0.01). There was no association with age, educational level, or diabetes medication (**Table 5**).

**Table 5.** Foot at-risk prevalence according to risk factors (regression analysis: crude and adjusted results)

Clinical characteristic	n/N	Foot at-risk prevalence (%)	p-value	Crude analysis			Adjusted analysis ^b^		
				PR	CI 95%	p-value	PR	CI 95%	p-value
Age									
< 60 years	86/178	48.3	0.034	1.00			1.00		
> 60 years	132/224	58.9		1.22	1.01-1.47	0.038	1.18	0.99-1.43	0.062
Gender									
Female	127/262	48.5	0.002	1.00			1.00		
Male	91/140	65.0		1.34	1.12-1.59	0.001	1.33	1.13-1.58	0.001
Educational level									
Illiterate	11/18	61.1	0.400	1.00					
Elementary	84/148	56.8		0.92	0.62-1.37	0.71			
High-school	105/194	54.1		0.89	0.60-1.30	0.54			
College	18 /42	42.9		0.70	0.42-1.16	0.17			
Diabetes duration									
< 10 years	114/239	47.7	0.001	1.00			1.00		
> 10 years	104/163	63.8		1.33	1.12-1.59	0.001	1.25	1.05-1.49	0.011
Diabetes medication									
Only diet	11/22	50.0	0.120	1.00					
Only oral antidiabetic drug	97/196	49.5		0.99	0.63-1.53	0.96			
Insulin	110/184	59.8		1.19	0.77-1.84	0.42			
Total Symptom Score									
Absent	42/95	44.2	0.02	1.00			1.00		
Mild (1-4.99)	110/204	53.9		1.22	0.94-1.58	0.13	1.18	0.91-1.52	0.20
Moderate-severe (>5)	66/103	64.1		1.44	1.11-1.90	0.007	1.40	1.07-1.82	0.012

**Legend:** ^a^Poisson regression with robust variance.

^b^Adjusted model to age, diabetes duration, gender, and Total Symptom Score. *Abbreviations:* PR -prevalence rate, CI -confidence interval.

## 4 Discussion

Our study revealed that more than half of outpatients (54.3%) were at risk of developing diabetic foot complications according to the new IWGDF 2019 criteria and were associated with severe Total Symptom Score, male sex, and diabetes duration, which corresponds to other cohort studies [17,26].

In the 2019 criteria, categories R1-R3 include PAD in addition and equivalently to PN. The inclusion of PAD in these categories increased the foot at-risk prevalence by 17% in our study. PAD was added to the guidelines 20 years after the first IWGDF criteria were established; this was done based on a systematic review of cohort studies of prognostic factors for ulceration [19]. We did not find any peer review studies with updated criteria yet.

Prevalence rates of foot at-risk evaluations using the 1999 criteria vary between 13% and 78% [6-13]. Factors that influence this wide range include reference population (general or hospital), local prevalence of diabetes, health system, modifications to the original criteria, and variability in the measurement of PN and PAD. For instance, a local general population study found a prevalence of 13% [13], while hospital- based studies found much higher frequencies because biomechanical deformity was defined as a moderate level of foot at-risk with no association with neuropathy, which raised the prevalence to 78% [11].

PN diagnosis by this classification (1999) does not include neuropathic symptoms. However, we found an association of foot at-risk with a higher Total Symptom Score (p=0.04). Generally, symptoms are classified as possible neuropathy using the Toronto criteria, although other conditions may also cause pain [27]. Performing two tests of long and short nerve fibers may increase the precision of neuropathy diagnosis. To confirm PN diagnosis, a nerve conduction test is required, but it is used only in case of doubt in daily clinical practice, and its use was not noted in the records available to us.

We found 16% of patients to have ABI > 1.3 in at least one artery. This finding points to the condition of arterial calcification, which is also called Monckeberg atherosclerosis, but it does not necessarily mean decreased blood flow. We could not classify these patients as having PAD because we did not have a second diagnostic method. Therefore, we did not include them in the PAD group, which may have resulted in the prevalence of PAD being underestimated. Exact PAD diagnosis requires additional methods such as brachial toe index, arterial Doppler ultrasonography, or invasive techniques [28]. Aboyans et al. reported that even subclinical PAD (ABI > 1.5) is associated with coronary artery disease (CAD) and should be considered a predictive condition of CAD [29].

Men were affected by foot at-risk more than woman, but women attended for consultation more frequently. Previous clinical studies have shown a higher prevalence of ulcer, re-ulceration, hospitalization, major amputation, and death in men than women [19,30]. Diabetes duration is frequently associated with poor glycemic and metabolic outcome [19]. Prolonged glycemic exposition of arteries in joints is associated with increased stiffness and affects the tibiotalar and hallux phalangeal metatarsal joint [31]. Therefore, they require close monitoring.

## 5 Public Health Implications

Our results reveal a hidden risk of diabetes complications in patients at risk of ulceration. Usually, local hospitals carry out screening programs for diabetes patients at risk of foot ulceration, but there are not enough health facilities that offer adequate preventive or therapeutic interventions [11,32]. A positive achievement at the government level was the development of a diabetic foot guide for primary care [33].

Although the foot at-risk diagnosis procedure is an easy-to-use, easily accessible, and non-invasive tool, it is not widely used and there is low physicians’ compliance [34]. Even if physicians diagnose PN or PAD, referrals to specialists are not carried out promptly in many cases [35], which may be due to a lack of understanding of ulceration or amputation and the infrequent occurrence of PN and PAD at the primary healthcare level [36]. The diagnosis could also be problematic at the community level because of the high prevalence of diabetes, limited consultation time, lack of evaluator’s training, or lack of necessary equipment for diagnosis [37].

Applying preventive and therapeutic measures to patients promptly according to their diabetic foot risk has been shown to reduce amputations by 48-78%, hospitalizations by 47-49%, and re-ulcerations by 48% in case series from the US and Europe [38]. Such measures are cost-effective and may even be applied in low income areas [39]. Prevention programs must be applied nationwide, and clinical guidelines give a strong recommendation for their application [40].

## 6 Limitations

The study’s limitation include the lack of laboratory tests, e.g. for HbAlc or lipid profile. Also, we did not evaluate other comorbidities that may contribute to foot at-risk, such as diabetic retinopathy or chronic kidney disease. Furthermore, the sample may not have been representative of the nationwide population as it represented only one of the less affluent areas of south Lima. Therefore, the results cannot be extrapolated to the entire country. Also, the diagnosis of arterial calcification (ABI > 1.3) needed a second reference test to define the degree of ischemia, either transcutaneous oxygen pressure or arterial wave form pulsatility, but their use was not noted in the records available to us. Finally, although the study did not aim to compare the classifications (1999 or 2019) in terms of better prediction of feet at risk of ulceration, we applied the updated 2019 definition to a population previously evaluated by the 1999 classification and showed how much the prevalence changed.

The strength of the study was that the foot at-risk program followed at the María Auxiliadora Hospital used a form which was validated by experts, created by endocrinologists, cardiovascular surgeons, and internists, and which used the IWGDF criteria as a reference. Also, PN diagnosis was made according to the Toronto consensus, and we observed a sufficiently large sample to achieve statistically robust results.

## 7 Conclusions

Our study revealed that there is a substantial burden of diabetic foot risk, in particular in men, elderly, patients with a long duration of diabetes, and those with painful neuropathy. The study also showed that the IWGDF 2019 criteria are helpful in revealing hidden foot at-risk cases. One out of two subjects with type 2 diabetes mellitus at the María Auxiliadora Hospital presented with a foot at risk of ulceration according to the updated guideline of the IWGDF 2019. The application of the 2019 criteria showed an increase of 16.9% compared with the previous definition.

More efforts are required at the primary care or hospital level to detect and treat this critical condition more reliably and promptly to avoid serious complications such as ulcerations and amputations. Likewise, reference centers with multidisciplinary teams are needed to apply preventive and therapeutic interventions. Finally, we recommend validating whether the updated 2019 definition better predicts the occurrence of ulcer compared to the previous 1999 definition, which requires a cohort study with a minimum 3-year follow-up to assess ulcer development. The present study may act as a baseline evaluation of a subsequent cohort study to assess ulcer development and to compare the predictive capability of both classification guidelines.

## Abbreviations

ABI: ankle brachial index

CAD: coronary artery disease

CI: confidence interval

IWGDF: international working group on diabetic foot

PAD: peripheral arterial disease

PN: peripheral neuropathy

PR: prevalence ratio
